# Diagnostic Odyssey in an Adult Patient with Ophthalmologic Abnormalities and Hearing Loss: Contribution of RNA-Seq to the Diagnosis of a PEX1 Deficiency

**DOI:** 10.3390/ijms232012367

**Published:** 2022-10-15

**Authors:** Gerard Muñoz-Pujol, Socorro Alforja-Castiella, Ricardo Casaroli-Marano, Blai Morales-Romero, Judit García-Villoria, Vicente A. Yépez, Julien Gagneur, Mirjana Gusic, Holger Prokisch, Frederic Tort, Antonia Ribes

**Affiliations:** 1 Secció d’Errors Congènits del Metabolisme-IBC, Servei de Bioquímica i Genètica Molecular, Hospital Clínic de Barcelona, IDIBAPS, CIBERER, 08028 Barcelona, Spain; 2 Department of Surgery, School of Medicine and Health Science, Universitat de Barcelona, Hospital Clínic de Barcelona, 08028 Barcelon, Spain; 3 Institute of Human Genetics, School of Medicine, Technical University of Munich, 81675 Munich, Germany; 4 Department of Informatics, Technical University of Munich, 85748 Garching, Germany; 5 Institute of Neurogenomics, Helmholtz Zentrum München, 85764 Neuherberg, Germany

**Keywords:** macular oedema, retinal dystrophy, sensorineural hearing loss, myopathic facies, *PEX1*, hypomorphic mutation, RNA-seq, very-long chain LPC

## Abstract

Peroxisomal biogenesis disorders (PBDs) are a heterogeneous group of genetic diseases. Multiple peroxisomal pathways are impaired, and very long chain fatty acids (VLCFA) are the first line biomarkers for the diagnosis. The clinical presentation of PBDs may range from severe, lethal multisystemic disorders to milder, late-onset disease. The vast majority of PBDs belong to Zellweger Spectrum Disordes (ZSDs) and represents a continuum of overlapping clinical symptoms, with Zellweger syndrome being the most severe and Heimler syndrome the less severe disease. Mild clinical conditions frequently present normal or slight biochemical alterations, making the diagnosis of these patients challenging. In the present study we used a combined WES and RNA-seq strategy to diagnose a patient presenting with retinal dystrophy as the main clinical symptom. Results showed the patient was compound heterozygous for mutations in *PEX1*. VLCFA were normal, but retrospective analysis of lysosphosphatidylcholines (LPC) containing C22:0–C26:0 species was altered. This simple test could avoid the diagnostic odyssey of patients with mild phenotype, such as the individual described here, who was diagnosed very late in adult life. We provide functional data in cell line models that may explain the mild phenotype of the patient by demonstrating the hypomorphic nature of a deep intronic variant altering *PEX1* mRNA processing.

## 1. Introduction

Peroxisomes are cell organelles that play an essential role in human metabolism and are indispensable for normal life [1]. Defects in genes encoding peroxisomal proteins may lead to a variety of diseases that can be grouped into two main classes, namely single peroxisomal enzyme deficiencies and peroxisome biogenesis disorders (PBDs).

In this report, we are going to focus on PBDs. These disorders are a heterogeneous group of autosomal recessive defects with a generalized impairment in peroxisome functioning. Peroxisomes are formed by fission following import of newly synthetized peroxisomal proteins from the cytoplasm into the peroxisome. Mutations in the genes encoding these proteins give rise to a peroxisomal protein import defect, which in turn leads to a deficiency of peroxisomal biogenesis. Moreover, proteins involved in maintenance and division of peroxisomes have also been described [1].

The clinical presentation of PBDs may range from severe, lethal multisystemic disorders to milder, late-onset progressive neurological disease or even isolated visual and/or hearing problems. Based on clinical presentation, three distinct subtypes are recognized among PBDs: the Zellweger spectrum disorders (ZSDs), rhizomelic chondrodysplasia punctate (RCDP) type 1 and type 5, and the peroxisomal fission defects [1].

The vast majority of PBDs belong to ZSDs that include the three historically defined clinical entities: cerebro-hepato-renal syndrome or Zellweger syndrome (ZS, OMIM 214100), neonatal adrenoleukodystrophy (NALD, OMIM 202370), and infantile Refsum disease (IRD, OMIM 266510), which are now considered different presentations within the same clinical and biochemical spectrum, and represents a continuum of overlapping clinical symptoms with ZS being the most and IRD the less severe disease [1,2,3,4,5,6]. Genetic studies showed that all ZSDs are caused by mutations in one of the PEX genes [6,7,8,9].

Because patients of ZSDs present a defect in peroxisome formation, multiple peroxisomal pathways are impaired, resulting in several metabolic abnormalities. These cell organelles carry out essential metabolic functions such as β-oxidation of very long-chain fatty acids (VLCFA), α-oxidation of phytanic acid, biosynthesis of plasmalogens and bile acids, and glyoxylate detoxification, among others [9,10,11]. Consequently, ZSDs patients accumulate very long chain fatty acids (VLCFA), phytanic and pristanic acid, C27-bile acid intermediates and pipecolic acid in plasma and have a deficiency of plasmalogens in erythrocytes, which are the first line biomarkers for the diagnosis [1,9,11].

In the past years, the implementation of next-generation sequencing (NGS) in the clinical setting has allowed the identification of an increasing number of mild clinical conditions in adult patients who frequently present normal or slight biochemical alterations in plasma [5,12,13,14,15,16,17,18,19]. To this regard, Heimler Syndrome (HS) has been recognized as a peroxisome biogenesis disorder within the ZSDs and added to the (very) mild end of the clinical spectrum. HS has been associated with mutations in *PEX1*, *PEX6* and *PEX26*. However, there are no significant differences among the gene-associated phenotypes [13,14,20,21,22,23]. 

Clinically, HS is characterized by sensorineural hearing loss, amelogenesis imperfecta of the teeth and retinal dystrophy [13,23,24]. In contrast to other ZSDs, this mild form shows no neurological manifestations and may present unaltered plasma VLCFA or other biochemical markers of PBDs [20]. The clinical heterogeneity of PBDs and the fact that mild forms often do not show biochemical alterations makes the diagnosis of these patients challenging. 

In the present study we used a combined WES and RNA-seq strategy in a patient presenting a mild clinical phenotype partially overlapping with HS, with retinal dystrophy as the main clinical symptom. Results showed the patient was compound heterozygous for mutations in the *PEX1* gene. VLCFA were normal, but retrospective analysis of lysosphosphatidylcholines (LPC) containing C22:0–C26:0 species was altered. We provide functional data in cell line models that explain the mild phenotype of the patient by demonstrating the hypomorphic nature of a deep intronic variant altering *PEX1* mRNA processing.

## 2. Results

### 2.1. Identification of PEX1 Mutations

A combined WES and RNA-seq strategy was performed to identify the underlying genetic defect in this family. WES was done in the index case and in her healthy parents, and data was analyzed following the filtering steps detailed in the methods section. We identified the c.1842del heterozygous variant in *PEX1* (NM_000466.3) in the index case, which was also present in her father. The variant was predicted to change a conserved glutamate to lysine at position 615 of the protein and generates a frameshift leading to a premature termination codon (p.Glu615LysfsTer30). The identified variant was considered to be pathogenic as it has been previously reported in patients with Zellweger syndrome [25,26], but WES could not identify any other variant in this gene, as it would be expected for an autosomal recessive disease. 

**Figure 1:**
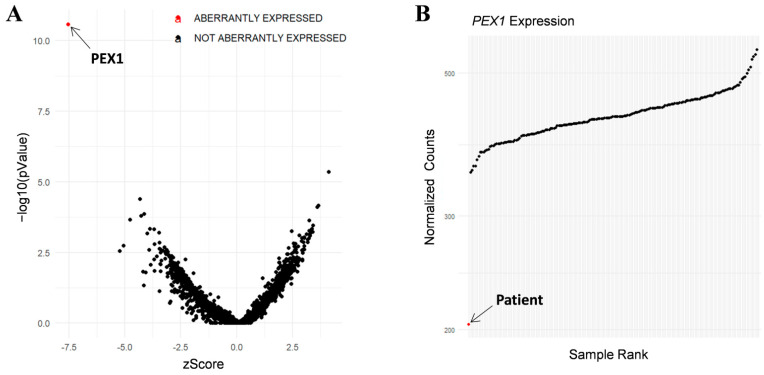
RNA-seq analysis in patient’s fibroblasts. (**A**) RNA expression volcano plot showing gene level significance (−log10 *p* value) against Z-scores. Red dot highlights *PEX1* as the most aberrantly expressed gene in this patient. (**B**) Expression Rank plot indicates (red dot) that the levels of *PEX1* mRNA levels detected in our patient were the lowest among the entire cohort of transcriptomes analyzed.

**Figure 2:**
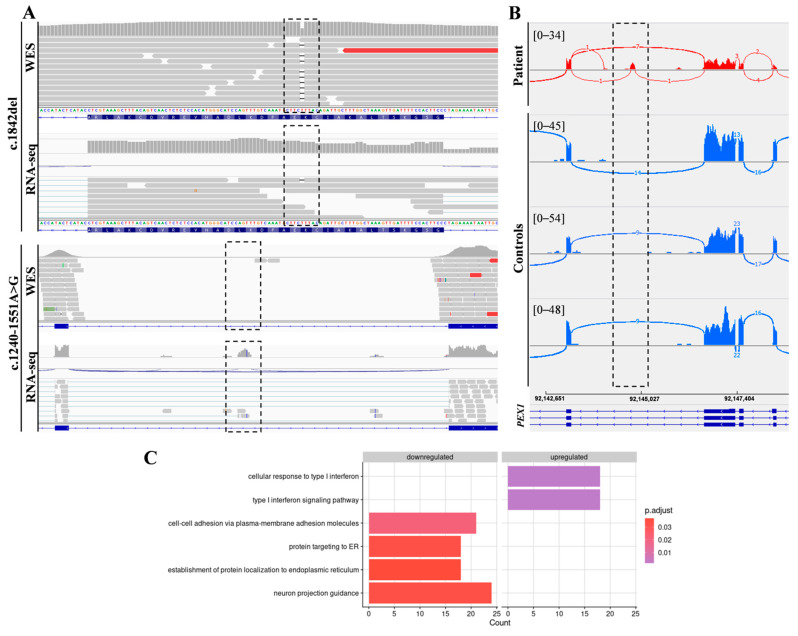
Combined WES and RNA-seq analysis identified *PEX1* heterozygous variants. (**A**) The analysis of DNA and RNA mapped reads identified two heterozygous variants in *PEX1* gene. The c.1842del variant was identified by WES, while the c.1240-1551A>G deep intronic substitution was not covered by WES but was captured by RNA-seq. (**B**) Sashimi plots showing the aberrant incorporation of an intronic sequence into *PEX1* mRNA in patient fibroblasts compared to controls. (**C**) Gene Ontology (GO) analysis of differentially expressed genes showed significantly enriched GO terms for the indicated biological processes.

In addition, RNA-seq was performed in the patient’s fibroblasts. Transcriptomic data were analyzed by running the DROP computational workflow in order to detect genes with aberrant expression levels, altered splicing events, and genes with mono-allelic expression (MAE) of rare variants. These analyses prioritized a statistically significant aberrant expression event involving *PEX1*, by showing a 46% reduction of the mRNA levels of this gene compared to controls. No other significant aberrant splicing or MAE outliers were prioritized. The levels of *PEX1* detected in our patient were the lowest of the cohort of transcriptomes analyzed (Figure 1). The analysis of the RNA mapped reads showed almost complete absence of the allele carrying the c.1842del variant identified by WES, suggesting that it may be mainly degraded by the nonsense mediated mRNA decay (NMD) mechanisms (Figure 2A). In addition, RNA-seq data analysis identified a 120 bp sequence of *PEX1* intron 5 that was aberrantly incorporated to mRNA between exons 5 and 6 (Figure 2A,B). Interestingly, this aberrant transcript was detected approximately in half of the reads, meaning that a remaining pool of correctly-spliced *PEX1* mRNA was still present in the patient’s cells. Remarkably, traces of aberrantly processed *PEX1* transcripts were also observed in several control individuals, suggesting the susceptibility of this region to be exonized. This intronic retention was predicted to generate a frameshift and a premature stop codon (p.Ile414GlnfsTer11). Transcriptome analysis also identified a deep intronic variant within the retained sequence (c.1240-1551A>G). This variant was not annotated in gnomAD. In silico analysis using Human Splicing Finder predicted no significant impact on splicing signals, and according to the American College of Medical Genetics (ACMG) criteria this variant was classified as a variant of uncertain significance (VUS) [27].

Segregation analysis of the identified variants confirmed the index case was compound heterozygous. The mother carried the c.1240-1551A>G variant, while the father carried the c.1842delA mutation. 

### 2.2. Functional Annotation Analysis of Differentially Expressed Genes

Functional annotation using gene ontology (GO) terms showed an enrichment in genes involved in the following categories: cell-cell adhesion via plasma-membrane adhesion molecules (21 genes), protein targeting to ER (18 genes), establishment of protein localization to endoplasmic reticulum (18 genes), neuron projection guidance (24 genes), cellular response to type I interferon (18 genes), and type I interferon signaling pathway (18 genes) (Figure 2C, Supplementary Table S1).

### 2.3. PEX1 cDNA Analysis

**Figure 3:**
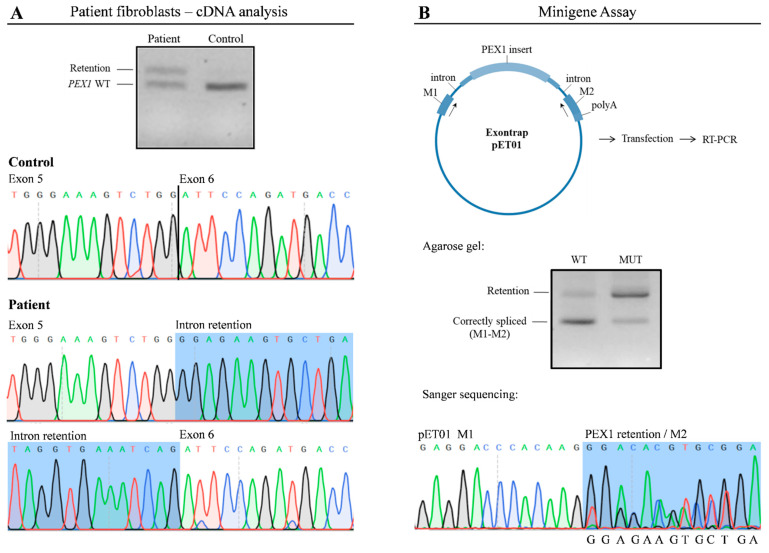
The c.1240-1551A>G variant leads to altered *PEX1* mRNA processing. (**A**) RT-PCR followedby agarose gel analysis and Sanger sequencing showed the exonization of a *PEX1* intronic sequence in patient’s fibroblasts. (**B**) A minigene assay was performed to determine functional impact of the c.1240-1551A>G variant. Wild type and mutant plasmids were transfected into HAP1 cells followed by RT-PCR. A predominant 200 bp product corresponding to correctly-spliced pET01 mRNA was detected upon transfection with wild type plasmids. In contrast, altered splicing was detected in cells transfected with the vector containing the c.1240-1551A>G variant, showing a significant overrepresentation of a 320 bp fragment. WT, wild type; MUT, mutant.

To corroborate the findings of RNA-seq, we analyzed *PEX1* mRNA by RT-PCR (Figure 3A); cDNA obtained from patient and control fibroblasts was amplified using specific primers. As expected, in control cells a 607 bp product was amplified, corresponding to the wild type *PEX1* cDNA, while in patient fibroblasts a higher molecular weight fragment in addition to the wild type product was obtained. Sanger sequencing demonstrated that this product corresponded to a *PEX1* fragment in which the 120 bp intronic sequence was incorporated. We also confirmed the presence of the c.1240-1551A>G variant within this fragment. Altogether, these results corroborate the observations obtained by transcriptomic analysis. 

### 2.4. The c.1240-1551A>G Enhances the Exonization of a PEX1 Intronic Sequence

To determine the pathogenicity of the c.1240-1551A>G deep intronic variant, we performed a functional assay using an minigene Exontrap vector system in which we cloned the wild type (pET01-WT) and mutant (pET01-MUT) *PEX1* genomic region (Figure 3B). HAP1 cells transfected with these constructs were analyzed by RT-PCR and Sanger sequencing using vector-specific primers. Cells transfected with pET01-WT produced a predominant 200 bp product which corresponded with correctly-spliced pET01 mRNA. In contrast, cells transfected with the pET01-MUT vector showed a significant overrepresentation of a 320 bp fragment containing the c.1240-1551A>G variant, despite the presence of residual amounts of correctly-spliced mRNA (Figure 3B). These results mimicked the observations made by RNA-seq and cDNA analysis, demonstrating that the presence of the c.1240-1551A>G mutation promotes the aberrant incorporation of an intronic sequence into *PEX1* mRNA.

## 3. Discussion

In the last decade, NGS technologies have emerged as a fundamental tool for the diagnosis of Mendelian disorders, especially those with wide phenotypic spectrum [28]. Regardless of the significant improvement provided by the implementation of WES, the genetic cause of the disease still remains to be identified in a significant number of patients. This is mainly due to difficulties in the interpretation and prioritization of candidate variants, and also to the fact that around 30% of disease-causing variants are located in non-coding regions, which are not covered by WES [29,30]. Recent studies highlight the usefulness of RNA-seq as a complementary diagnostic tool [31,32,33,34]. Indeed, the development of appropriate bioinformatic tools for RNA-seq data analysis, such as DROP, allows the prioritization of candidate disease-associated genes by detecting aberrantly expressed genes, altered splicing events and monoallelic expression of rare variants [34]. 

In the case we present here, the use of RNA-seq was decisive to prioritize the disease causing gene and uncover the pathomechanism underlying the disorder. The clinical presentation of our patient was compatible with Heimler-like syndrome, which has been previously associated to recessive *PEX1*, *PEX6*, and *PEX26* mutations [20,22]. Importantly, in addition to hearing loss, retinal dystrophy and enamel abnormalities, typically reported in HS individuals, our patient showed dysmorphic features, usually observed in affected individuals with PBDs but never documented in HS [13,20,23,24]. Macular edema was only reported in few HS patients, and to our knowledge neutropenia has never been associated to this syndrome. In spite of that, our first suspicion was a peroxisomal disorder. Consequently, VLCFAs and phytanic acid in plasma were measured, but all parameters were within the control range. Because of the lack of peroxisomal abnormalities in plasma and the clinical overlap with Usher syndrome (MIM: 276900), the differential diagnosis with this disease was also considered. However, WES analysis did not reveal any mutation in Usher associated genes, but identified a single nucleotide deletion within *PEX1* coding region (c.1842del, p.Glu615LysfsTer30), which has been previously reported as pathogenic in patients with ZS [25,26]. This finding motivated the search for the second mutation. Therefore, RNAseq studies were undertaken. Results showed a significant reduction of *PEX1* expression and identified a deep intronic substitution (c.1240-1551A>G) on this gene. This variant was not covered by WES and was detected exclusively by RNA-seq, which showed a mixture of *PEX1* aberrant transcripts that retained an intronic sequence containing this substitution, together with a remaining pool of correctly spliced mRNA. Although this alteration was predicted to generate a frameshift leading to a truncated protein product (p.Ile414GlnfsTer11), the transcriptomic data suggested that the aberrant transcripts may escape—at least partially—the NMD degradation system. In silico analysis of the c.1240-1551A>G variant predicted no significant impact on splicing and was classified as VUS [27]. Therefore, functional studies were mandatory to fully demonstrate its contribution to the patient’s phenotype.

Minigene assay showed that HAP1 cells transfected with a vector carrying the c.1240-1551A>G variant had a marked increase of aberrantly-spliced transcripts compared to cells transfected with the wild type plasmid. Our functional data mimicked the observations made in patient’s fibroblasts and, on the basis of these findings, we concluded that the mRNA processing defect observed in the patient was caused the by c.1240-1551A>G variant. It is of note that a small proportion of aberrantly-spliced transcripts were also detected in cells expressing the wild type vector. Indeed, the detailed analysis of the RNA-seq mapped reads in our control cohort also showed traces of aberrant *PEX1* transcripts carrying the retention of this genomic region in several controls, suggesting susceptibility of this region to be exonized. Altogether, the transcriptomic and functional data strongly suggest that the c.1240-1551A>G substitution is pathogenic by contributing to the activation of a cryptic splice site signal that enhances exonization of this intronic sequence. 

In addition, RNA-seq analysis of differentially expressed genes showed an enrichment of genes associated to endoplasmic reticulum (ER), neuron guidance and interferon response. These results are consistent with observations made in PBD-derived iPSCs and *PEX1* knock-in mouse where ER and some interferon pathway associated genes were also significantly enriched [35,36]. Moreover, our results support the proposed hypothesis that peroxisome and ER function are strikingly linked [37]. 

*PEX1* loss of function mutations are usually associated to ZS, while genotypes harboring less severe variants are more frequently found in other milder peroxisomal phenotypes. In the case of HS, the mild phenotype of the patients was explained by the presence of at least one hypomorphic allele [20]. Indeed, complementation studies have shown that the proteins encoded by hypomorphic alleles retained residual activity and cause a mild peroxisomal dysfunction. However, functional evidence has been provided only in a limited number of patients [20]. The patient reported here is compound heterozygous for the c.1842del loss of function mutation and the newly identified c.1240-1551A>G variant. Our functional data demonstrated the hypomorphic nature of the c.1240-1551A>G variant. We showed that in addition to aberrantly-processed transcripts, a remaining pool of correctly-spliced *PEX1* mRNA was detected in the patient’s fibroblasts, as well as in the cell models expressing this mutation. These studies suggest that PEX1 function may be partially preserved in this individual and may explain the unaltered levels of VLCFA in serum. However, when we retrospectively analyzed the corresponding very long chain LPC, in the same serum sample, its elevation was evident, indicating a greater sensitivity of this methodology, as it has been pointed out by several authors [4,23,38,39,40]. Therefore, we strongly support the analysis of very long chain LPC when facing with a suspicion of a peroxisomal disorder. This simple test could avoid the diagnostic odyssey of patients with mild phenotype, such as that of the individual described here, who was diagnosed very late in the life, while initial symptoms appeared at three years of age. 

## 4. Materials and Methods

### 4.1. Case Report

A 42-year-old woman, from healthy non-consanguineous parents, was first referred with ophthalmological suspicion of retinal dystrophy at 28 years of age after suffering an anxiety attack when she experienced an episode of nyctalopia. Her parents told us that since childhood she always walked while holding onto her companions. The patient was using hearing aids due to bilateral sensorineural hearing loss diagnosed at three years of life, and erroneously attributed to previous treatment with ototoxic antibiotics. During her first decade, she underwent two surgical procedures for convergent strabismus. Physical and ophthalmological examination showed low visual acuity, sensorineural hearing loss, enamel abnormalities and subtle dysmorphic facial features, namely myopathic facies and palpebral fissure with lower temporal slant. Persistent neutropenia was also detected. 

**Figure 4:**
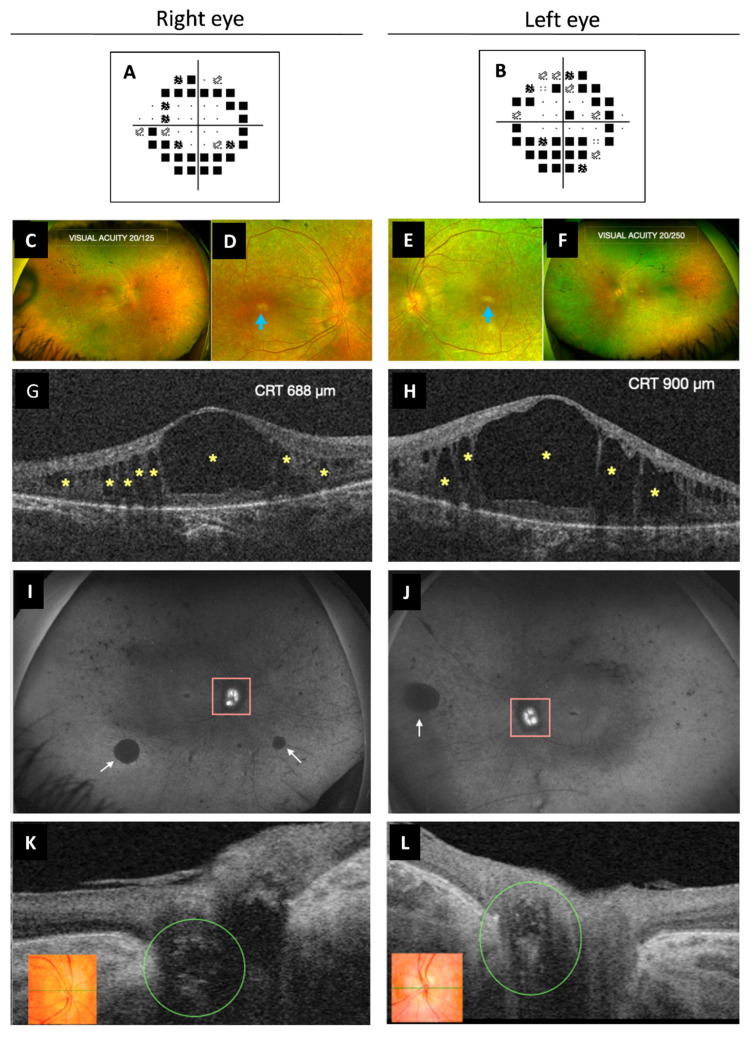
Multimodal imaging study of ophthalmological findings. (**A**,**B**) Automated campimetry revealed a concentric reduction in both eyes. (**C**–**F**) UWF fundus retinographies show clear vitreous, retinal vessel narrowing more pronounced in the peripheral retina, bilateral RPE atrophy, subtle pigmentary changes with some bone spicules and mottled pigment. It can be observed in more detail the poorly defined appearance of the optic disc (RE more than OS) due to ONH drusen and the whitish foveal appearance secondary to RPE atrophy (images **D**,**E**, blue arrow). (**G**,**H**) OCT exhibited cystoid macular oedema with large central and smaller surrounding hypo-reflective spaces (yellow asterisks). (**I**,**J**) UWF FAF images show ONH (pink square) hyper-FAF (bright white) due to the presence of ONH drusen and multiple areas of hypo-FAF secondary to RPE atrophy, such as the peripapillary area. Diffuse peripheral retinal mottling and some larger areas (white arrows), as well as the macular area, also exhibit hypo-FAF. (**K**,**L**) OCT showed optic disc drusen as ovoid hypo-reflective structures in both eyes (green circle). Color retinography detail of ONH (inserts **K**,**L**). UWF, ultra-widefield; RPE, retinal pigment epithelium; RE, right eye; OS, left eye; ONH, optic nerve head; FAF, fundus autofluorescence; OCT, optical coherence tomography; CRT, central retinal thickness.

Best corrected visual acuity was 20/100 in both eyes. Intraocular pressure was always normal, pachymetry was 609/606 µm for her right eye (RE) and left eye (OS), respectively, and campimetry revealed bilateral annular concentric defect (Figure 4A,B). Fundus examination showed optic disc drusen and vascular narrowing mostly in the periphery of the retina. Irregular patchy greyish and pigmentary changes in the form of bone spicules and mottling were demonstrated in the mid and far periphery of the retina (Figure 4C–F). Spectral-domain optical coherence tomography (OCT) showed macular oedema (ME) (Figure 4G,H) that was first treated with topical brinzolamide and oral acetazolamide. However, due to the chronic state of the ME, an intravitreal implant of 0.7 mg of dexamethasone (Ozurdex^®^, Allergan, Inc., Irvine, CA, USA) in the OS was applied. The patient refused other intravitreal treatments. For that reason, oral acetazolamide and topic brinzolamide drops were continued, with only partial resolution of the edema. Fundus autofluorescence showed peripapillary hypo-autofluorescence and retinal hypo-autofluorescent areas secondary to retinal atrophy, which showed increasing extension over time (Figure 4I,J), as well as the foveal hypo-autofluorescent area. Optic disc exhibited hyper-autofluorescence due to optic nerve head drusen. Swept-source OCT also showed drusen in the optic disc in both eyes (Figure 4K,L).

During the following years, she experienced progressive visual acuity deterioration, in part due to the persistence of ME and retinal dystrophy progression, and cataract appearance in OS. Phacoemulsification with intraocular lens implant in OS was performed. Ultra-widefield imaging allowed us to compare the distribution, extent and progression of all the parameters (foveal atrophy, vascular narrowing, peripapillary and retinal atrophy and pigmentary changes) already detected at the first visit. The patient has been followed up for 15 years. Last ophthalmological evaluation, six months ago, showed a best corrected visual acuity of 20/125 and 20/250 in RE and OS, respectively. However, she showed greater difficulties and insecurity in carrying out daily activities and was unable to move around independently. 

A peroxisomal disorder was suspected, but very long chain fatty acids (VLCFA and phytanic acid in plasma were normal making peroxisomal disorder unlikely. The second suspected disease was Usher syndrome (MIM: 276900), but genomic studies did not confirm this hypothesis but rather a mutation in *PEX1* that later on led to the diagnosis of PEX1 defect when the second mutation was identified by RNAseq analysis. Interestingly, retrospective study of very-long chain LPC (tandem mass spectrometry homemade methodology, using Perkin–Elmer kit) demonstrated alteration of these lipid species: C26:0-LPC, 0.48 µmol/L (C.V. ≤ 0.17); C24:0-LPC, 0.69 µmol/L (C.V. ≤ 0.27); C22:0-LPC, 0.28 µmol/L (C.V. ≤ 0.19). Of interest, this patient has recently been included in a large cohort study of Mendelian disorders [34]. In the present manuscript, we provide details on ophthalmologic abnormalities, biochemical data and functional studies demonstrating the pathogenic effect of the variants.

### 4.2. Whole Exome Sequencing

Whole exome sequencing and RNA-seq were performed in the Centre Nacional d’Anàlisi Genòmica (CNAG-CRG). The primary data files (FASTQ files) were analyzed using the pipeline developed by CNAG-CRG [41]. Sequence reads were mapped to Human genome build hg19/GRCh37. Trio-WES primary data was analyzed using the URDCAT genome-phenome analysis platform (https://rdcat.cnag.crg.eu/, accesed on 2 November 2021), filtered by frequency (allele frequency < 1% in population databases, including 1000G and gnomAD), and by functional impact on the encoded protein, as well as for the clinical and biochemical phenotype of the patients. 

### 4.3. RNA Sequencing

RNA-seq was performed in CNAG-CRG. The quality control of the total RNA was done using the Qubit^®^ RNA HS Assay (Life Technologies, Carlsbad, CA,USA) and RNA 6000 Nano Assay on a Bioanalyzer 2100 (Agilent Technologies, Santa Clara, CA, USA). Libraries were prepared using the TruSeq^®^ Stranded mRNA LT Sample Prep Kit (Illumina Inc., San Diego, CA, USA). The libraries were sequenced on a HiSeq 2500 (Illumina, San Diego, CA, USA) in paired-end mode (2 × 76 bp). Primary data analysis, image analysis, base calling, and quality scoring of the run were processed using the manufacturer’s software Real Time Analysis (RTA 1.18.66.3), followed by generation of FASTQ sequence files. Reads from RNA-seq were demultiplexed and then mapped with STAR (v2.7.0a) to the hg19 genome assembly. Analysis of the aligned data was done using DROP in order to detect aberrantly expressed genes, altered splicing events, and monoallelic expression (MAE) of rare variants [42,43,44]. Aberrant expression was detected using OUTRIDER, a statistical method that normalizes gene expression counts using a denoising autoencoder and fits a mean and dispersion for each sample-gene combination using a negative binomial distribution [42]. Z-scores are computed using the normalized counts. As controls for detection of aberrant expression and splicing, the cohort of 269 fibroblasts from patients with Mendelian disorders described in Yepez et al. 2020 was used.

Gene enrichment was performed on the significantly (OUTRIDER’s *p* < 0.05) down and up regulated genes (N = 593 and 748, respectively) using the enrichGO function from the clusterProfiler package in R (https://www.liebertpub.com/doi/10.1089/omi.2011.0118, accessed on 8 October 2022). Only biological processes were analyzed. Multiple testing across the different GO terms was performed using Benjamini–Hochberg’s method. Redundant terms were removed using the simplify function from the same package and a similarity cutoff = 0.7. Significant GO terms are those non-redundant with adjusted *p*-value < 0.05.

### 4.4. Cell Culture and cDNA Analysis

Skin derived fibroblasts obtained from the patient and control individuals were maintained in minimum essential medium (MEM) (1 g/L glucose, 10% fetal calf serum and 1% penicillin-streptomycin). Cells were grown to confluence in 25 cm^2^ flasks, harvested by trypsinization and pelleted by centrifugation or reseed. Total RNA extraction was performed using RNeasy kit (74104 Qiagen, Hilden, Germany) and single-stranded complementary DNA (cDNA) synthesized using oligodT primers and M-MLV Reverse Transcriptase, RNase H Minus, Point Mutant (Promega, Madison, WI, USA) according to the manufacturer’s protocols. *PEX1* cDNA was amplified by polymerase chain reaction (PCR) using self-designed oligonucleotides (available on demand) and analyzed by Sanger sequencing. 

### 4.5. Functional Studies

A minigene assay was performed to determine the effect of the *PEX1* c.1240-1551A>G variant. Briefly, we cloned a 281 bp genomic region containing the c.1240-1551A>G variant, as well as the wild type allele into the Exontrap Cloning Vector pET01 (MoBiTec GmbH, Göttingen, Germany). To test the effect of the c.1240-1551A>G variant on mRNA processing, we transfected the wild-type (pET01-WT) and mutant (pET01-MUT) plasmids into HAP1 cells using Lipofectamine3000 (Promega, Madison, WI, USA), following manufacturer’s instructions. Total RNA obtained 48 h upon transfection was subjected to reverse transcription-PCR (RT-PCR) using primers corresponding to the 5′ and 3′ pET01 exons (available on demand). The products were analyzed in agarose gels, followed by Sanger sequencing.

## 5. Conclusions

We report a patient with *PEX1* bi-allelic mutations presenting very mild biochemical alterations and a mild clinical form of ZSDs. We provide a molecular explanation for the mild phenotype of the patient demonstrating a leaky splicing defect. We also emphasize the usefulness of combined RNA-seq and functional validation studies to achieve definitive and reliable diagnoses in clinically heterogeneous disorders. In addition, we strongly recommend very long chain LPC to be added to the list of tests when a diagnosis of peroxisomal disorder is suspected.

## Data Availability

The data that support the findings of this study are available from the corresponding author, upon reasonable request.

## Supplementary Materials

The following supporting information can be downloaded at: https://www.mdpi.com/article/10.3390/ijms232012367/s1.

## References

[1] Waterham H.R., Ferdinandusse S., Wanders R.J. (2016). Human disorders of peroxisome metabolism and biogenesis. Biochim. Biophys. Acta.

[2] Gould S.J., Raymond G.V., Valle D., Scriver C.R., Beaudet A.L., Sly W.S., Valle D. (2001). The Peroxisomal Biogenesis Disorder.

[3] Steinberg S.J., Dodt G., Raymond G.V., Braverman N.E., Moser A.B., Moser H.W. (2006). Peroxisome biogenesis disorders. Biochim. Biophys. Acta.

[4] Klouwer F.C., Berendse K., Ferdinandusse S., Wanders R.J., Engelen M., Poll-The B.T. (2015). Zellweger spectrum disorders: Clinical overview and management approach. Orphanet J. Rare Dis..

[5] Braverman N.E., Raymond G.V., Rizzo W.B., Moser A.B., Wilkinson M.E., Stone E.M., Steinberg S.J., Wangler M.F., Rush E.T., Hacia J.G. (2016). Peroxisome biogenesis disorders in the Zellweger spectrum: An overview of current diagnosis, clinical manifestations, and treatment guidelines. Mol. Genet. Metab..

[6] Waterham H.R., Ebberink M.S. (2012). Genetics and molecular basis of human peroxisome biogenesis disorders. Biochim. Biophys. Acta.

[7] Braverman N.E., D’Agostino M.D., Maclean G.E. (2013). Peroxisome biogenesis disorders: Biological, clinical and pathophysiological perspectives. Dev. Disabil. Res. Rev..

[8] Fujiki Y., Okumoto K., Mukai S., Honsho M., Tamura S. (2014). Peroxisome biogenesis in mammalian cells. Front. Physiol..

[9] Wanders R.J.A., Baes M., Ribeiro D., Ferdinandusse S., Waterham H.R. (2022). The physiological functions of human peroxisomes. Physiol. Rev..

[10] Klouwer F.C., Huffnagel I.C., Ferdinandusse S., Waterham H.R., Wanders R.J., Engelen M., Poll-The B.T. (2016). Clinical and Biochemical Pitfalls in the Diagnosis of Peroxisomal Disorders. Neuropediatrics.

[11] Wanders R.J.A. (2018). Peroxisomal disorders: Improved laboratory diagnosis, new defects and the complicated route to treatment. Mol. Cell. Probes.

[12] Enns G.M., Ammous Z., Himes R.W., Nogueira J., Palle S., Sullivan M., Ramirez C. (2021). Diagnostic challenges and disease management in patients with a mild Zellweger spectrum disorder phenotype. Mol. Genet. Metab..

[13] Gao F., Hu F., Xu P., Qi Y., Li J., Zhang Y. (2019). Expanding the clinical and genetic spectrum of Heimler syndrome. Orphanet J. Rare Dis..

[14] Smith C.E., Poulter J.A., Levin A.V., Capasso J.E., Price S., Ben-Yosef T., Sharony R., Newman W.G., Shore R.C., Brookes S.J. (2016). Spectrum of PEX1 and PEX6 variants in Heimler syndrome. Eur. J. Hum. Genet..

[15] Renaud M., Guissart C., Mallaret M., Ferdinandusse S., Cheillan D., Drouot N., Muller J., Claustres M., Tranchant C., Anheim M. (2016). Expanding the spectrum of PEX10-related peroxisomal biogenesis disorders: Slowly progressive recessive ataxia. J. Neurol..

[16] Barillari M.R., Karali M., Di Iorio V., Contaldo M., Piccolo V., Esposito M., Costa G., Argenziano G., Serpico R., Carotenuto M. (2020). Mild form of Zellweger Spectrum Disorders (ZSD) due to variants in *PEX1*: Detailed clinical investigation in a 9-years-old female. Mol. Genet. Metab. Rep..

[17] Fujiki Y., Abe Y., Imoto Y., Tanaka A.J., Okumoto K., Honsho M., Tamura S., Miyata N., Yamashita T., Chung W.K. (2020). Recent insights into peroxisome biogenesis and associated diseases. J. Cell Sci..

[18] Nava E., Hartmann B., Boxheimer L., Capone Mori A., Nuoffer J.M., Sargsyan Y., Thoms S., Rosewich H., Boltshauser E. (2022). How to Detect Isolated PEX10-Related Cerebellar Ataxia?. Neuropediatrics.

[19] Berendse K., Engelen M., Ferdinandusse S., Majoie C.B., Waterham H.R., Vaz F.M., Koelman J.H., Barth P.G., Wanders R.J.A., Poll-The B.T. (2016). Zellweger spectrum disorders: Clinical manifestations in patients surviving into adulthood. J. Inherit. Metab. Dis..

[20] Ratbi I., Falkenberg K., Sommen M., Al-Sheqaih N., Guaoua S., Vandeweyer G., Urquhart J., Chandler K., Williams S., Roberts N. (2015). Heimler Syndrome Is Caused by Hypomorphic Mutations in the Peroxisome-Biogenesis Genes PEX1 and PEX6. Am. J. Hum. Genet..

[21] Ratbi I., Jaouad I.C., Elorch H., Al-Sheqaih N., Elalloussi M., Lyahyai J., Berraho A., Newman W.G., Sefiani A. (2016). Severe early onset retinitis pigmentosa in a Moroccan patient with Heimler syndrome due to novel homozygous mutation of PEX1 gene. Eur. J. Med. Genet..

[22] Neuhaus C., Eisenberger T., Decker C., Nagl S., Blank C., Pfister M., Kennerknecht I., Müller-Hofstede C., Charbel Issa P., Heller R. (2017). Next-generation sequencing reveals the mutational landscape of clinically diagnosed Usher syndrome: Copy number variations, phenocopies, a predominant target for translational read-through, and *PEX26* mutated in Heimler syndrome. Mol. Genet. Genom. Med..

[23] Daich Varela M., Jani P., Zein W.M., D’Souza P., Wolfe L., Chisholm J., Zalewski C., Adams D., Warner B.M., Huryn L.A. (2020). The peroxisomal disorder spectrum and Heimler syndrome: Deep phenotyping and review of the literature. Am. J. Med. Genet. Part C Semin. Med. Genet..

[24] Heimler A., Fox J.E., Hershey J.E., Crespi P. (1991). Sensorineural hearing loss, enamel hypoplasia, and nail abnormalities in sibs. Am. J. Med. Genet..

[25] Yik W.Y., Steinberg S.J., Moser A.B., Moser H.W., Hacia J.G. (2009). Identification of novel mutations and sequence variation in the Zellweger syndrome spectrum of peroxisome biogenesis disorders. Hum. Mutat..

[26] Ebberink M.S., Mooijer P.A., Gootjes J., Koster J., Wanders R.J., Waterham H.R. (2011). Genetic classification and mutational spectrum of more than 600 patients with a Zellweger syndrome spectrum disorder. Hum. Mutat..

[27] Desmet F.O., Hamroun D., Lalande M., Collod-Béroud G., Claustres M., Béroud C. (2009). Human Splicing Finder: An online bioinformatics tool to predict splicing signals. Nucleic Acids Res..

[28] Wright C.F., FitzPatrick D.R., Firth H.V. (2018). Paediatric genomics: Diagnosing rare disease in children. Nat. Rev. Genet..

[29] Boycott K.M., Ardigó D. (2018). Addressing challenges in the diagnosis and treatment of rare genetic diseases. Nat. Rev. Drug Discov..

[30] Stenson P.D., Mort M., Ball E.V., Evans K., Hayden M., Heywood S., Hussain M., Phillips A.D., Cooper D.N. (2017). The Human Gene Mutation Database: Towards a comprehensive repository of inherited mutation data for medical research, genetic diagnosis and next-generation sequencing studies. Hum. Genet..

[31] Kremer L.S., Bader D.M., Mertes C., Kopajtich R., Pichler G., Iuso A., Haack T.B., Graf E., Schwarzmayr T., Terrile C. (2017). Genetic diagnosis of Mendelian disorders via RNA sequencing. Nat. Commun..

[32] Lee H., Huang A.Y., Wang L., Yoon A.J., Renteria G., Eskin A., Signer R.H., Dorrani N., Nieves-Rodriguez S., Wan J. (2020). Diagnostic utility of transcriptome sequencing for rare Mendelian diseases. Genet. Med..

[33] Stenton S.L., Prokisch H. (2020). The Clinical Application of RNA Sequencing in Genetic Diagnosis of Mendelian Disorders. Clin Lab Med..

[34] Yépez V.A., Gusic M., Kopajtich R., Mertes C., Smith N.H., Alston C.L., Ban R., Beblo S., Berutti R., Blessing H. (2022). Clinical implementation of RNA sequencing for Mendelian disease diagnostics. Genome Med..

[35] Demaret T., Roumain M., Ambroise J., Evraerts J., Ravau J., Bouzin C., Bearzatto B., Gala J.L., Stepman H., Marie S. (2020). Longitudinal study of Pex1-G844D NMRI mouse model: A robust pre-clinical model for mild Zellweger spectrum disorder. Biochimica et biophysica acta. Mol. Basis Dis..

[36] Wang X.M., Yik W.Y., Zhang P., Lu W., Huang N., Kim B.R., Shibata D., Zitting M., Chow R.H., Moser A.B. (2015). Induced pluripotent stem cell models of Zellweger spectrum disorder show impaired peroxisome assembly and cell type-specific lipid abnormalities. Stem Cell Res. Ther..

[37] Smith J.J., Aitchison J.D. (2013). Peroxisomes take shape. Nat. Rev. Mol. Cell Biol..

[38] Ventura M.J., Wheaton D., Xu M., Birch D., Bowne S.J., Sullivan L.S., Daiger S.P., Whitney A.E., Jones R.O., Moser A.B. (2016). Diagnosis of a mild peroxisomal phenotype with next-generation sequencing. Mol. Genet. Metab. Rep..

[39] Klouwer F.C.C., Ferdinandusse S., van Lenthe H., Kulik W., Wanders R.J.A., Poll-The B.T., Waterham H.R., Vaz F.M. (2017). Evaluation of C26:0-lysophosphatidylcholine and C26:0-carnitine as diagnostic markers for Zellweger spectrum disorders. J. Inherit. Metab. Dis..

[40] Jaspers Y.R.J., Ferdinandusse S., Dijkstra I.M.E., Barendsen R.W., van Lenthe H., Kulik W., Engelen M., Goorden S.M.I., Vaz F.M., Kemp S. (2020). Comparison of the Diagnostic Performance of C26:0-Lysophosphatidylcholine and Very Long-Chain Fatty Acids Analysis for Peroxisomal Disorders. Front. Cell Dev. Biol..

[41] Laurie S., Fernandez-Callejo M., Marco-Sola S., Trotta J.R., Camps J., Chacón A., Espinosa A., Gut M., Gut I., Heath S. (2016). From Wet-Lab to Variations: Concordance and Speed of Bioinformatics Pipelines for Whole Genome and Whole Exome Sequencing. Hum. Mutat..

[42] Brechtmann F., Mertes C., Matusevičiūtė A., Yépez V.A., Avsec Ž., Herzog M., Bader D.M., Prokisch H., Gagneur J. (2018). OUTRIDER: A Statistical Method for Detecting Aberrantly Expressed Genes in RNA Sequencing Data. Am. J. Hum. Genet..

[43] Mertes C., Scheller I.F., Yépez V.A., Çelik M.H., Liang Y., Kremer L.S., Gusic M., Prokisch H., Gagneur J. (2021). Detection of aberrant splicing events in RNA-seq data using FRASER. Nat. Commun..

[44] Yépez V.A., Mertes C., Müller M.F., Klaproth-Andrade D., Wachutka L., Frésard L., Gusic M., Scheller I.F., Goldberg P.F., Prokisch H. (2021). Detection of aberrant gene expression events in RNA sequencing data. Nat. Protoc..

